# Transcriptomic and Metabolomic Profiling Reveals Differential Responses of Soybean Germination to Neutral and Alkaline Salt Stresses

**DOI:** 10.3390/biology15090670

**Published:** 2026-04-24

**Authors:** Yujie Jin, Lijun Pan, Dingkun Qian, Yuntian Zhao, Shengbo Xu, Hongtian Wang, Zhuo Zhang, Jian Wei

**Affiliations:** 1 Plant Biotechnology Center, College of Agronomy, Jilin Agricultural University, Changchun 130118, China; 20230757@mails.jlau.edu.cn (Y.J.); 20252037@mails.jlau.edu.cn (L.P.); 20240722@mails.jlau.edu.cn (D.Q.); 20240733@mails.jlau.edu.cn (Y.Z.); 20230760@mails.jlau.edu.cn (S.X.); 20230770@mails.jlau.edu.cn (H.W.); 2 Northern Key Laboratory of Saline-Tolerant Soybean Breeding, Ministry of Agriculture and Rural Affairs, Faculty of Agronomy, Jilin Agricultural University, Changchun 130118, China

**Keywords:** soybean, salt stress, alkali stress, seed germination, transcriptomics, metabolomics

## Abstract

Saline–alkali stress severely limits crop production worldwide. Soybean [*Glycine max* (L.) Merr.] is particularly sensitive during seed germination, a stage critical for stand establishment. This complex stress environment encompasses two distinct yet equally critical dimensions: neutral salt stress and alkaline salt stress, each eliciting specialized physiological and metabolic responses. Here, a comparative assessment of four genotypes (tolerant: CN16, CN17; sensitive: Williams 82, K18) under 100 mmol/L Na^+^ revealed that alkaline salt stress exerts a significantly more potent inhibitory effect than neutral salt stress. Tolerant cultivars maintained 75–80% germination under alkaline conditions, whereas sensitive ones dropped below 15%, a divergence primarily driven by superior oxidative mitigation capacity. Integrated multi-omics analysis of the tolerant variety CN16 identified stage-specific regulatory shifts: early alkaline salt stress (60 h) triggers extensive transcriptional reprogramming focused on physical barrier reinforcement, including cell walls and lipid remodeling. By 96 h, regulatory modes between the two stress types diverged: neutral salt elicited a transcriptional surge, while alkaline salt transitioned to a metabolically dominant regulation, shifting flux from growth-related isoflavonoids to defense-related anthocyanins. Crucially, this study uncovers the distinct bioenergetic trade-offs governing these responses: whereas adaptation to neutral salt relies on low-energy galactose metabolism, tolerance to alkaline salt demands energy-intensive processes, specifically the active vacuolar compartmentalization of organic acids and anthocyanins for intracellular buffering. This obligatory energy expenditure restricts biomass accumulation, explaining the severe growth penalties observed in complex saline-alkali environments. Finally, the identification of a core regulatory module driven by key genes, including *GmPHOT2b*, *GmLOG*, and *GmSHMT08*, enriches the metabolic regulatory network under saline-alkali stress, providing core targets and a theoretical framework for precisely balancing metabolic expenditure with biomass accumulation in breeding practice.

## Simple Summary

Soil salinization and alkalization significantly reduce global soybean yields, with seed germination being the most vulnerable stage. This study investigated why alkaline salt inhibits soybean germination more severely than neutral salt. By comparing four soybean varieties, we found that tolerant varieties maintained high germination rates under alkaline conditions, whereas sensitive varieties were severely suppressed. Advanced molecular analysis revealed that the biological costs of adapting to these two stresses differ fundamentally. Adaptation to neutral salt relies on sugar metabolism to maintain water balance, which consumes relatively less energy. In contrast, adaptation to alkaline salt requires the synthesis of organic acids and active proton transport to neutralize the high-pH environment. This process consumes a large amount of biological energy, thereby reducing the resources available for plant growth. We also identified specific genes that regulate these processes. These findings provide a theoretical basis and genetic targets for breeding new soybean varieties capable of growing in complex saline–alkali soils.

## 1. Introduction

Soybean [*Glycine max* (L.) Merr.] is a pivotal global source of vegetable protein and oil. Given the widening gap between supply and demand, the utilization of marginal lands—particularly saline-alkali soils—has emerged as a strategic imperative to ensure food security. However, soil salinization and alkalization pose severe constraints on agricultural sustainability. According to recent assessments by the Food and Agriculture Organization of the United Nations (FAO), salt-affected soils are globally widespread, with their severity and extent being exacerbated by climate change and improper irrigation practices [1,2]. Saline-alkali conditions severely compromise soybean yield and quality by deteriorating soil physicochemical properties and inhibiting nutrient acquisition [3]. Consequently, elucidating the mechanisms underlying soybean saline-alkali tolerance and identifying elite genetic resources are fundamental prerequisites for enhancing agricultural productivity on such marginal lands.

Soil saline environments are rarely uniform; they naturally manifest as two distinct categories, neutral and alkaline salts, which impose fundamentally different phytotoxic mechanisms on plants. Neutral salt stress (predominantly NaCl) inhibits plant growth mainly through the dual effects of osmotic stress and ion toxicity. High salinity induces physiological dehydration, while excessive Na^+^ accumulation triggers membrane lipid peroxidation and a reactive oxygen species (ROS) burst, ultimately disrupting metabolic homeostasis [4,5]. In contrast, alkaline salt stress, typified by sodium bicarbonate (NaHCO_3_), often exerts more severe lethal effects [6,7]. Mechanistically, this stress involves the superimposition of high-pH stress and specific carbonate and bicarbonate (CO_3_^2−^/HCO_3_^−^) toxicity onto basal osmotic and Na^+^ stress factors. Such combined stress not only results in nutrient deprivation by inducing the precipitation of key elements (e.g., Fe, Zn, and P) in the rhizosphere but also disrupts the transmembrane proton motive force due to high environmental pH, thereby substantially increasing the energetic cost for plants to buffer intracellular acid-base changes [8,9,10]. Such a fundamental mechanistic disparity underscores that alkaline salt stress is not merely a simple superimposition of neutral salt stress. Therefore, theoretical frameworks derived solely from neutral salt models are insufficient to accurately elucidate the actual adaptive mechanisms of soybean in complex alkaline environments.

Research on plant salt tolerance mechanisms has established the ion homeostasis theory, which centers on the SOS signaling pathway [11] and the NHX family [12]. Building upon this foundation, significant breakthroughs have been achieved in identifying key soybean salt tolerance genes, particularly in the domains of ion transport and transcriptional regulation. In terms of structural genes, Guan et al. [13] identified the major locus *GmSALT3* for seedling salinity tolerance using map-based cloning, elucidating its mechanism in limiting root-to-shoot Na^+^ transport. Additionally, Do et al. [13] verified the function of the allele *Ncl* at the reproductive stage, demonstrating that it regulates Cl^−^ homeostasis and reduces yield loss in saline fields. Regarding transcriptional regulation, Zhou et al. [14] and Wang et al. [15] focused on seedling stress resistance. They characterized members of the *GmWRKY* family (e.g., *GmWRKY21*) and *GmMYB84*, respectively. Through stress-induced expression analysis, they revealed that these transcription factors enhance tolerance by activating downstream antioxidant enzyme systems and regulating the synthesis of osmolytes such as proline. However, these classical studies were predominantly conducted under single neutral salt (NaCl) stress conditions and focused primarily on the vegetative seedling or reproductive stages. Seed germination, representing the inception of the plant life cycle, involves a unique transition from heterotrophy to autotrophy that is accompanied by the mobilization of seed reserves and hormonal remodeling [16]. Kan et al. [17] confirmed, via dual analysis of natural and mapping populations, that salt tolerance during germination shows no significant correlation with seedling performance. Moreover, tolerance at this stage is governed by independent genetic loci that are distinct from classical seedling tolerance mechanisms, such as *GmSALT3* on chromosome 3. Germination acts as the critical checkpoint that determines final stand establishment in saline fields, yet it faces physiological challenges fundamentally different from those at the seedling stage. As a result, theoretical frameworks derived solely from seedling models cannot fully capture this complexity. Thus, characterizing how soybean coordinates ionic and metabolic signals to differentiate its responses to neutral versus alkaline salt stress during this specific window is scientifically imperative to complete the comprehensive framework of salt tolerance.

Given that saline-alkali tolerance in plants is a complex quantitative trait controlled by multiple genes, and that the germination stage involves extensive energy metabolic responses and signal network remodeling, traditional single-gene functional validation is often insufficient to capture the full landscape of the regulatory network. Integrated transcriptomic and metabolomic analysis provides a systemic perspective ranging from gene transcription to metabolites, directly linking upstream regulatory signals with downstream biochemical phenotypes. As a result, it has become a powerful tool for unraveling complex stress resistance mechanisms [18]. For instance, Jin et al. [19] utilized a combined transcriptomic and metabolomic strategy to investigate soybean root salt tolerance, dissecting the response network under NaCl stress. This study revealed the synergistic regulatory mechanism involving the phenylpropanoid metabolic pathway and ion transporters. In parallel, Fu et al. [20] employed an integrated multi-omics approach encompassing transcriptomics, proteomics, and metabolomics to deeply explore the molecular responses of cultivated soybean seedlings to NaCl stress. They elucidated that cutin and wax biosynthesis, along with flavonoid accumulation, represent key adaptive mechanisms for coping with high-salinity environments. However, these representative studies focused predominantly on single neutral salt (NaCl) stress conditions and were restricted to the seedling stage or root tissues. Currently, a systematic comparative multi-omics analysis of cultivated soybean germination under strict equimolar Na^+^ concentrations (neutral salt versus alkaline salt) is essential to fully decouple the effects of high pH from ion toxicity. Without such an integrated analysis, the specific mechanisms by which plants orchestrate ion homeostasis, intracellular buffering, and metabolic responses to adapt to these two distinct habitats during this specific window remain to be elucidated. Hence, exploiting multi-omics technologies to identify common and specific regulatory hubs and key metabolic markers represents a pivotal avenue for deconstructing the molecular network of soybean saline-alkali tolerance during germination.

To address this scientific imperative, the present study focuses on the critical stage of soybean germination. We established neutral salt (NaCl) and alkaline salt (NaHCO_3_) stress treatments with strict equimolar Na^+^ concentrations. By employing an integrated transcriptomic and metabolomic analysis, we systematically investigated the differential responses and molecular regulatory networks of soybean under these two distinct saline-alkali conditions. Specifically, the objectives of this study were to (i) characterize the phenotypic variations and physiological limiting factors of soybean germination under different saline-alkali stress regimes; (ii) deconstruct the common and specific response mechanisms involving ion homeostasis, intracellular buffering processes, and metabolic networks in response to neutral and alkaline stresses; and (iii) identify key candidate genes and metabolic markers regulating saline-alkali tolerance. This study provides novel insights into the molecular networks of soybean saline-alkali tolerance from a multidimensional perspective and offers promising molecular targets for breeding new soybean varieties adapted to complex saline-alkali environments.

## 2. Materials and Methods

### 2.1. Plant Materials and Growth Conditions

Based on the evaluation system established by Han et al. [21], an extensive preliminary screening of soybean germplasm resources preserved at Jilin Agricultural University (Changchun, China) was conducted using principal component analysis (PCA) and the membership function method. Consequently, two soybean cultivars exhibiting superior saline-alkali tolerance during germination, CN16 and CN17, and two sensitive accessions, Williams 82 (W82) and K18, were selected as experimental materials. Prior to the experiment, healthy, plump, and intact seeds of uniform size were selected. Seeds were surface-sterilized with 75% ethanol for 30 s, rinsed three times with deionized water, and blotted dry with sterile filter paper. Subsequently, the seeds were uniformly placed in 9 cm Petri dishes lined with two layers of filter paper (20 seeds per dish), and 10 mL of treatment solution was added to each dish. All seeds were incubated in a growth chamber under controlled conditions: a 15 h light/9 h dark photoperiod, a day/night temperature cycle of 28/24 °C, and 60–70% relative humidity. Treatment solutions were renewed every 24 h to maintain constant ion concentrations.

### 2.2. Experimental Design and Stress Treatments

To evaluate the differential effects of saline and alkaline stresses while strictly controlling for ion concentration, a completely randomized design was employed using four soybean cultivars (CN16, CN17, W82, and K18). The experiment consisted of three treatment groups with equimolar Na^+^ concentrations: (1) control group (CK), treated with deionized water; (2) neutral salt group (SS), treated with 100 mmol/L sodium chloride (NaCl, Sinopharm Chemical Reagent Co., Ltd., Shanghai, China); (3) alkaline salt group (SA), treated with 100 mmol/L sodium bicarbonate (NaHCO_3_, Sinopharm Chemical Reagent Co., Ltd., Shanghai, China).

Germination was monitored every 12 h following the start of incubation. A seed was defined as germinated when the radicle protruded through the seed coat and reached half the length of the seed. Germination parameters were calculated in accordance with the standard protocols of the International Seed Testing Association (ISTA) and the methods proposed by Kader [22]. The germination percentage (GP) was calculated to evaluate the final germination capacity as follows:

(1) $$GP=\frac{n_{t}}{N}\times 100\%$$

where n_t_ represents the cumulative number of germinated seeds at time t, and N is the total number of tested seeds. The Germination Increment (GI_inc_) was calculated to determine the proportion of newly germinated seeds within each time interval using the equation:

(2) $$GI_{inc}=\frac{n_{i}}{N}\times 100\%$$

where n_i_ represents the number of newly germinated seeds within the time interval (12 h), and N is the total number of tested seeds (20 seeds).

**Figure 1:**
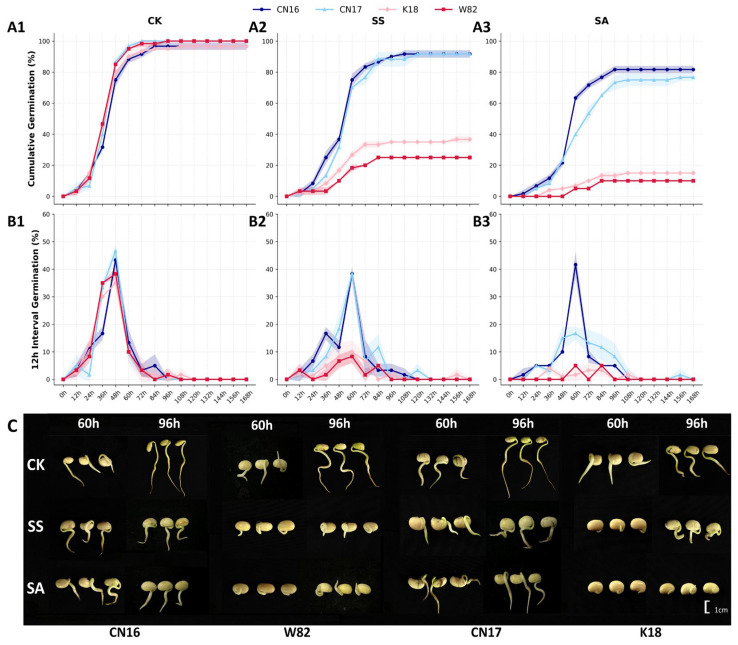
Germination dynamics and seedling phenotypes of four soybean cultivars under neutral and alkaline-salt stresses. (**A1**–**A3**) Time course of cumulative germination percentage (GP) over a 168 h experimental period. (**B1**–**B3**) Germination increment (GI_inc_) measured at 12 h intervals. (**C**) Representative photographs of seedling morphology at 60 h and 96 h. Data points in (**A1**–**A3**,**B1**–**B3**) represent the mean (*n* = 3). Tested cultivars include tolerant (CN16, CN17) and sensitive (W82, K18) genotypes.

To identify the optimal sampling window, critical time points were determined by integrating the germination dynamics of the tolerant cultivar CN16 with the soybean seedling developmental staging system [23]: (i) 60 h (active germination phase), corresponding to the G2–G3 stages (rapid elongation of the hypocotyl-radicle axis and root hair development). At this point, GI_inc_ peaked (Figure 1(B1)), indicating maximal metabolic activity and transcriptional regulation. This represents the optimal window for analyzing early stress responses. A (ii) 96 h time point (stable germination phase) corresponded to the G4 stage (development of lateral root primordia). At this stage, GP approached saturation (Figure 1(A1)), and seedling morphogenesis was largely complete, primarily reflecting the adaptive physiological state under prolonged stress.

Based on these critical time points, uniform sampling was conducted for all cultivars at 60 h and 96 h. At the time of sampling, seed coats were removed, and embryonic axes were collected, flash-frozen in liquid nitrogen, and stored at −80 °C. Experimental sample allocation was as follows: (1) morphological and physiological measurements were performed on all four cultivars with three biological replicates. To ensure representative sampling and minimize individual variation, each biological replicate consisted of pooled embryonic axes collected from at least 10 randomly selected seedlings grown in an independent Petri dish. (2) Multi-omics analysis samples were selected only from CN16 due to its superior tolerance. Specifically, three biological replicates were used for transcriptomic sequencing, and six biological replicates were used for untargeted metabolomics analysis.

### 2.3. Physiological and Biochemical Assays

To evaluate the osmotic adjustment capacity and redox status of soybean germination under saline-alkali stress, embryonic axes from the four cultivars (CN16, CN17, W82, and K18) were sampled for physiological index measurements.

#### 2.3.1. Osmotic Adjustment Substances, Oxidative Damage Indices, and CAT Activity

The contents of proline (Pro), malondialdehyde (MDA), hydrogen peroxide (H_2_O_2_), superoxide anion ($O_{2}^{-}$), and the activity of catalase (CAT) was determined using commercial assay kits (Sangon Biotech, Shanghai, China) following the manufacturer’s instructions. Briefly, approximately 0.1 g of fresh embryonic axis tissue was weighed, homogenized in the extraction buffer provided in the kit, and centrifuged to obtain the supernatant. Pro content was measured using the acid ninhydrin colorimetric method at 520 nm. MDA content was determined via the thiobarbituric acid (TBA) method based on absorbance at 532 nm and 600 nm. H_2_O_2_ content was assayed using the titanic complex method at 415 nm. The $O_{2}^{-}$ content was measured based on the hydroxylamine oxidation method at 530 nm. CAT activity was assayed by monitoring the decomposition of H_2_O_2_ at 240 nm.

#### 2.3.2. Antioxidant Enzyme Activity

Enzyme extraction and activity assays were performed following the method of Pan et al. [24] with minor modifications, rather than using commercial kits. All physiological and biochemical indices were calculated relative to fresh weight (FW).

Superoxide Dismutase (SOD) Activity: Fresh samples (0.2 g) were homogenized on ice in pre-cooled phosphate buffer (50 mmol/L, pH 7.8) and centrifuged to collect the supernatant. Activity was determined using the nitroblue tetrazolium (NBT, Shanghai Macklin Biochemical Co., Ltd., Shanghai, China) photochemical reduction method [25]. The reaction mixture was exposed to 4000 lux illumination. One unit of enzyme activity (U) was defined as the amount of enzyme required to inhibit NBT photochemical reduction by 50%.

Peroxidase (POD) Activity: Fresh samples (0.2 g) were homogenized on ice in pre-cooled phosphate buffer (100 mmol/L, pH 6.0), and the supernatant was collected after centrifugation. Activity was measured using the guaiacol (Shanghai Macklin Biochemical Co., Ltd., Shanghai, China) colorimetric method [26]. Enzyme activity was calculated by monitoring the change in absorbance at 470 nm over 1 min. One unit of enzyme activity (U) was defined as an absorbance change of 0.01 per minute.

#### 2.3.3. Determination of Electrolyte Leakage

Electrolyte leakage (EL), a standard physiological indicator of cell membrane integrity and stress-induced damage, was evaluated by measuring the relative electrical conductivity. Fresh embryonic axis samples (approximately 0.1 g) were washed three times with deionized water to remove surface electrolytes. The samples were then immersed in glass tubes containing 10 mL of deionized water and incubated at 25 °C for 2 h on a shaker. The electrical conductivity of the deionized water alone was measured as the blank background (C_0_). The initial electrical conductivity (C_1_) of the sample solution was measured using a DDS-307 conductivity meter (Leici, Shanghai, China). Subsequently, the tubes were boiled in a water bath for 20 min to completely rupture the cell membranes and release all intracellular electrolytes. After cooling to room temperature, the final electrical conductivity (C_2_) was recorded. The relative electrical conductivity (REC) was calculated using the following formula:

REC (%) = (C_1_ − C_0_)/(C_2_ − C_0_) × 100. (3)

### 2.4. Transcriptome Sequencing and Analysis

To elucidate the transcriptional reprogramming mechanisms underlying saline-alkali tolerance, embryonic axis samples from the superior tolerant cultivar CN16 (collected at 60 h and 96 h) were used for cDNA library construction and transcriptome sequencing. Total RNA was extracted from the treated samples using the RNAprep Pure Plant Kit (TIANGEN, Beijing, China) according to the manufacturer’s instructions. RNA quality and integrity were assessed using a NanoDrop 2000 spectrophotometer (Thermo Fisher Scientific, Waltham, MA, USA) and an Agilent 2100 Bioanalyzer (Agilent Technologies, Santa Clara, CA, USA) prior to library construction. Sequencing was performed on an Illumina NovaSeq platform (Illumina, San Diego, CA, USA) (PE150 mode), generating at least 5.8 Gb of clean data per sample with a Q30 score > 96%.

After removing adapters and low-quality sequences, clean reads were mapped to the soybean reference genome (*Glycine max* Wm82.gnm2) using HISAT2 v2.1.0 [27]. Subsequently, transcripts were assembled using StringTie 2.2.1 [28], and di fferential expression analysis was conducted using DESeq2 1.40.2 [29]. The thresholds for identifying differentially expressed genes (DEGs) were set as $|\log_{2}FoldChange|\geq 1$ and FDR < 0.01.

To dissect the common and specific response mechanisms under salt and alkali stresses, DEGs identified from four pairwise comparisons (CK60 vs. SS60, CK60 vs. SA60, CK96 vs. SS96, and CK96 vs. SA96) were categorized into four characteristic sets: (a) early common response set, comprising genes co-regulated by SS and SA at 60 h; (b) late common response set, comprising genes co-regulated by SS and SA at 96 h; (c) alkali-specific temporal set, comprising genes consistently responsive at both 60 h and 96 h solely under SA treatment; and (d) salt-specific temporal set, comprising genes consistently responsive at both 60 h and 96 h solely under SS treatment. Functional enrichment analysis of the identified DEGs was performed using the GO [30] and KEGG [31] databases. A Benjamini–Hochberg adjusted *p*-value (*P*adj < 0.05) served as the threshold for significant enrichment.

### 2.5. Untargeted Metabolomics Analysis

In parallel with the transcriptome analysis, the same embryonic axis samples from cultivar CN16 were used for untargeted metabolomics profiling.

#### 2.5.1. Metabolite Extraction

Approximately 100 mg of sample powder, ground in liquid nitrogen, was weighed and mixed with the extraction solvent prior to ultrasonic extraction. Following centrifugation, the supernatant was collected, evaporated to dryness under vacuum, and reconstituted for instrumental analysis. Chromatographic separation was performed using an ACQUITY UPLC HSS T3 column (1.8 µm, 2.1 mm × 100 mm, Waters, Milford, MA, USA) coupled with a Xevo G2-XS QTOF high-resolution mass spectrometer (Waters, Milford, MA, USA) for data acquisition in both positive and negative ionization modes. Quality Control (QC) samples were interspersed throughout the run at regular intervals to monitor system stability and data reproducibility.

#### 2.5.2. Data Preprocessing and Identification of Differentially Accumulated Metabolites

Raw mass spectrometry data were processed using Progenesis QI software 3.0 (Waters, Milford, MA, USA) for peak alignment, peak picking, and normalization to generate a peak intensity matrix. Following log_2_ transformation and unit variance (UV) scaling, orthogonal partial least squares discriminant analysis (OPLS-DA) models were constructed using the ropls package 1.32.0 in R 3.4.1 to identify discriminating features between groups [32]. Variable importance in projection (VIP) values were extracted from the models to quantify the contribution of metabolites, and model reliability was assessed via permutation tests. The screening criteria for differentially accumulated metabolites (DAMs) were set as VIP > 1 and Student’s *t*-test *p* < 0.05.

#### 2.5.3. Screening Strategy for Core Metabolites

Metabolites were categorized into corresponding sets (a–d) following the same logic as the gene sets described in Section 2.4. To minimize false positives and identify the most robust biological markers, a multi-step screening approach was applied. First, an initial threshold of variable importance in projection (VIP) > 1 and Student’s *t*-test *p* < 0.05 was used to capture the broad stress-responsive metabolic pool. Candidate metabolites within each set were then ranked in descending order based on their maximum absolute $\left|log_{2}FoldChange\right|$ values across the comparison groups. To isolate features with the most substantial biological effect sizes, only the top 20 metabolites from each set were selected for Z-score normalization and heatmap visualization. Subsequently, the top 20 metabolites from all sets were pooled. To ensure absolute quantitative reliability, any metabolite with missing or incomplete quantitative data across the biological replicates was strictly excluded. The final screened core metabolites were subjected to two-way hierarchical clustering analysis. Heatmaps were plotted based on Z-score normalized data, calculated using the formula:

(4) $$Z=\frac{x-\mu }{\sigma }$$

where x is the raw intensity of the metabolite, μ is the mean intensity of the row, and σ is the standard deviation of the row.

### 2.6. Weighted Gene Co-Expression Network Analysis (WGCNA)

A weighted gene co-expression network was constructed based on gene expression levels (FPKM) using the WGCNA package 1.7.1 in R 3.4.1 [33]. A soft-thresholding power (β) was determined to ensure that the network conformed to the scale-free topology criterion. Co-expression modules were identified using the Dynamic Tree Cut algorithm based on the topological overlap matrix (TOM). The minimum module size was set to 30, and the cut height for module merging was set to 0.25. To identify core regulatory modules, Pearson correlation analysis was performed between module eigengenes (MEs) and a pre-selected representative panel of core physiological indices (Pro, MDA, H_2_O_2_, SOD, and POD). These five specific indices were chosen as network anchors because they independently represent the three primary physiological dimensions of stress adaptation: osmotic adjustment, oxidative damage, and basal antioxidant defense, while avoiding statistical redundancy from highly collinear variables. Modules exhibiting significant correlations (*p* < 0.05) with these core traits were identified as key response modules, from which hub genes were subsequently screened.

### 2.7. Joint Transcriptome and Metabolome Analysis

Differentially expressed genes (DEGs) and differentially accumulated metabolites (DAMs) were mapped to the Kyoto Encyclopedia of Genes and Genomes (KEGG) database. Pathway enrichment analyses were conducted independently for transcriptomic and metabolomic datasets using Fisher’s exact test. *p*-values were adjusted via the Benjamini–Hochberg method to yield false discovery rates (*Q*_DEG_ and *Q*_DAM_). Key regulatory pathways were identified by retaining only those that exhibited significant enrichment (*Q* < 0.05) in at least one dataset and simultaneously contained both DEGs and DAMs. Subsequently, these pathways were ranked based on their combined enrichment significance. This ranking score was calculated as the sum of the negative log-transformed *Q*-values from both datasets. This metric prioritized the most significant metabolic pathways for subsequent analysis. To further quantify the direct regulatory relationships between the transcriptomic and metabolomic layers beyond canonical pathways, a Pearson correlation analysis was performed. The FPKM values of the 5 validated transcriptomic hub genes (derived from WGCNA) and the relative abundances of the 19 core stress-responsive metabolites across the six experimental groups were extracted and subjected to Pearson correlation coefficient (*r*) calculations. Significant correlations were identified based on the criteria of |*r*| > 0.8 and *p* < 0.05. The comprehensive correlation matrix, highlighting these significant interactions, was visualized as a heatmap.

### 2.8. Quantitative Real-Time PCR (qRT-PCR) Analysis

To validate the reliability of the transcriptome data, key differentially expressed genes (DEGs) were selected for qRT-PCR analysis. The quality of extracted total RNA was assessed using a Unano-1000 micro-spectrophotometer (Unano, Hangzhou, China). Only samples with an A_260_/A_280_ ratio between 2.0 and 2.2 were used for subsequent analysis. First-strand cDNA was synthesized using the TRUEscript 1st Strand cDNA Synthesis Kit (Aidlab, Beijing, China) following the manufacturer’s instructions. qRT-PCR assays were performed using an FQD-96C Real-Time Quantitative Thermal Cycler (Bioer Technology, Hangzhou, China). Gene-specific primers were designed using Primer3 software 2.6.0, with soybean (*Glycine max*) 18S rRNA serving as the internal reference gene. The experiment included three biological replicates, and relative expression levels were calculated using the 2^−ΔΔC_t_^ method [34].

### 2.9. Statistical Analysis

All experimental data are presented as the mean ± standard deviation (SD) calculated from three independent biological replicates. Statistical analyses were performed using IBM SPSS Statistics 27.0 (IBM Corp., Armonk, NY, USA). To evaluate the significance of differences among different treatments within the same cultivar at specific time points, data were analyzed using one-way analysis of variance (ANOVA) followed by Duncan’s multiple range test. A *p*-value of <0.05 was considered to indicate a statistically significant difference. All figures were generated using GraphPad Prism 9.0 (GraphPad Software, San Diego, CA, USA), Python 3.10 (Python Software Foundation, Wilmington, DE, USA) and the R 3.4.1 (R Core Team, Vienna, Austria) statistical environment.

## 3. Results

### 3.1. Morphological, Biochemical, and Physiological Changes in Soybean Seeds During Germination Under Saline-Alkali Stress

#### 3.1.1. Genotype-Specific Responses of Seed Germination Under Saline-Alkali Stress

Comparative analysis of germination dynamics under equimolar Na^+^ concentrations revealed that alkaline salt stress exerted a significantly stronger inhibitory effect on germination than neutral salt stress, with significant variations in tolerance observed among the cultivars (Figure 1). In the control group (CK), germination trends were remarkably consistent across the four cultivars, evidenced by highly overlapping cumulative germination percentage (GP) curves. Germination increments (GI_inc_) were concentrated between 36 and 48 h, indicating robust seed vigor (Figure 1(A1,B1)).

Under neutral salt treatment (SS), the peak germination period for all cultivars was delayed to approximately 60 h. Despite this delay, the final GP of tolerant cultivars (CN16 and CN17) remained above 90%, whereas that of sensitive cultivars (K18 and W82) was significantly inhibited, plateauing at approximately 40% (Figure 1(A2,B2)). Under alkaline salt treatment (SA), inter-cultivar disparities were further exacerbated. CN16 and CN17 maintained a final GP of 75–80%, demonstrating strong adaptability. Notably, the GI_inc_ of CN16 at 60 h was significantly higher than that of CN17, indicating faster initiation during the early stress phase (Figure 1(A3,B3)). In contrast, the germination processes of K18 and W82 were nearly stagnant; only sporadic germination was observed, with final rates remaining below 15% (Figure 1(A3,B3)). These trends were consistent with morphological observations. In the alkaline environment, sensitive cultivars primarily exhibited failed radicle protrusion or necrosis and browning of the radicle tip (Figure 1C).

#### 3.1.2. Selection of Critical Sampling Time Points

To accurately dissect the mechanisms underlying saline-alkali tolerance, the germination dynamics of the superior tolerant cultivar CN16 were used as a benchmark to pinpoint two critical time windows for standardized physiological and omics sampling. As shown in Figure 1(B1), the germination increment (GI_inc_) of CN16 peaked significantly at 60 h. This time point corresponded to the G2–G3 developmental stages (rapid elongation of the hypocotyl and radicle), characterizing the most active phase of metabolic reactivation and transcriptional regulation. Thus, 60 h was established as the optimal window for analyzing early initiation responses.

Subsequently, the cumulative germination percentage (GP) curve reached a stable plateau at 96 h (Figure 1(A1)). This marked the completion of seedling morphogenesis and the transition to the G4 stage, reflecting steady-state adaptation under prolonged stress. Based on these critical time points derived from the tolerant germplasm, parallel physiological and biochemical measurements were conducted on all four cultivars to compare genotype-specific responses. Furthermore, an in-depth integrated transcriptomic and metabolomic analysis was performed specifically on CN16 to unravel the core molecular networks underpinning its superior tolerance.

#### 3.1.3. Seedling Morphogenesis and Physiological/Biochemical Indices During Germination

**Figure 2:**
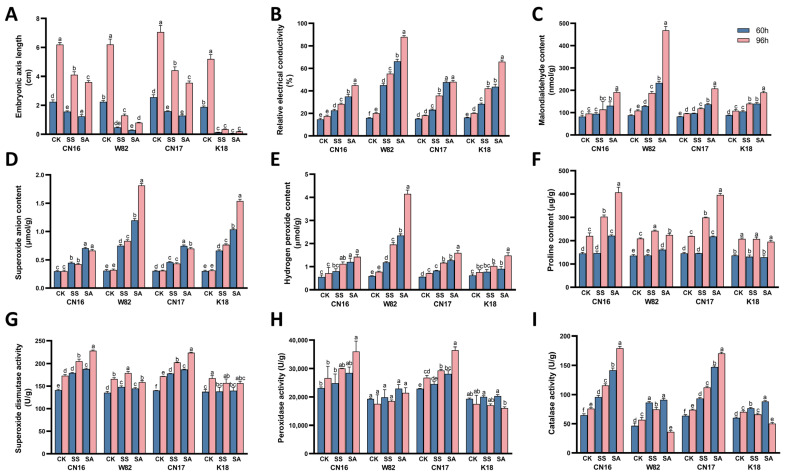
Embryonic axis elongation and physiological and biochemical responses of soybean seedlings under neutral and alkaline salt stresses: (**A**) seedling hypocotyl length; (**B**) relative electrical conductivity (REC); (**C**) malondialdehyde (MDA) content; (**D**) superoxide anion ($O_{2}^{-}$) content; (**E**) hydrogen peroxide (H_2_O_2_) content; (**F**) proline (Pro) content; (**G**) superoxide dismutase (SOD) activity; (**H**) peroxidase (POD) activity; and (**I**) catalase (CAT) activity. Data are presented as means ± SD (*n* = 3). Different lowercase letters above the bars indicate statistically significant differences between treatments at *p* < 0.05, according to Duncan’s multiple range test. Bars sharing the same letter are not significantly different. CK: Control (deionized water); SS: Neutral salt stress; SA: Alkaline salt stress. Samples were collected at 60 h and 96 h. CN16 and CN17: genotypes tolerant to saline and alkaline stress; W82 and K18: genotypes sensitive to saline and alkaline stress.

Measurements of embryonic axis length revealed that seedlings in the control group elongated continuously at both 60 h and 96 h (Figure 2A). In contrast, embryonic axis elongation in both stress treatment groups was inhibited to varying degrees, with the inhibitory effect of alkaline salt stress significantly exceeding that of neutral salt stress. Under alkaline salt treatment (SA), the embryonic axis lengths of the tolerant cultivars CN16 and CN17 were significantly greater than those of sensitive cultivars at both sampling points. Notably, at 96 h, the growth of the sensitive cultivars K18 and W82 was severely inhibited, showing a marked reduction in length compared to the control group. Conversely, tolerant cultivars exhibited relatively less inhibition and maintained a certain degree of elongation.

Analysis of cell membrane integrity and reactive oxygen species (ROS) accumulation (Figure 2B–E) revealed that relative electrical conductivity (REC), MDA, superoxide anion ($O_{2}^{-}$), and H_2_O_2_ contents increased significantly in all cultivars following SA treatment. However, the extent of oxidative damage differed markedly between genotypes. In sensitive cultivars, both ROS generation, specifically $O_{2}^{-}$ and H_2_O_2_, and membrane damage indicators, such as REC and MDA, accumulated continuously, reaching their highest levels at the late stress stage of 96 h. Conversely, tolerant cultivars effectively restricted the excessive accumulation of ROS and maintained significantly lower levels of REC and MDA under the same alkaline stress, demonstrating superior cell membrane stability.

Measurements of osmolytes and antioxidant enzyme activities (Figure 2F–I) further elucidated the divergent defense capacities. Under severe alkaline stress, the activities of core ROS-scavenging enzymes, including SOD, POD, and CAT, in tolerant cultivars were significantly induced and robustly maintained at high levels throughout the 96 h period. Additionally, these tolerant cultivars exhibited substantial proline accumulation. In contrast, although sensitive cultivars showed initial increases in antioxidant enzyme activities, their SOD, POD, and CAT activities failed to be maintained and showed a significant decline during the late stress stage of 96 h, falling significantly below those of the tolerant cultivars. Correspondingly, their proline accumulation remained significantly lower throughout the treatment period.

### 3.2. Transcriptome Profiling and Functional Annotation of Differentially Expressed Genes (DEGs) in the Tolerant Cultivar

To elucidate the transcriptional response of the tolerant cultivar CN16 to saline-alkali stress, transcriptome sequencing was performed on embryonic axis samples collected at 60 h and 96 h. Each sample yielded 5.8–6.5 Gb of clean data, with Q30 values exceeding 96% (Supplementary Table S1). The number of identified DEGs exhibited significant temporal specificity (Table 1). At 60 h, SA treatment induced 4754 DEGs (2386 upregulated and 2368 downregulated), whereas SS treatment induced only 1553 DEGs (736 upregulated and 817 downregulated). By 96 h, the number of DEGs under SA treatment decreased to 1103 (727 upregulated and 376 downregulated), while those under SS treatment surged to 10,783 (4786 upregulated and 5997 downregulated). These results indicate that the transcriptional response to SA was concentrated in the early germination stage with balanced regulation, whereas the SS response was more pronounced in the later stage, characterized by a bias toward gene repression.

**Figure 3:**
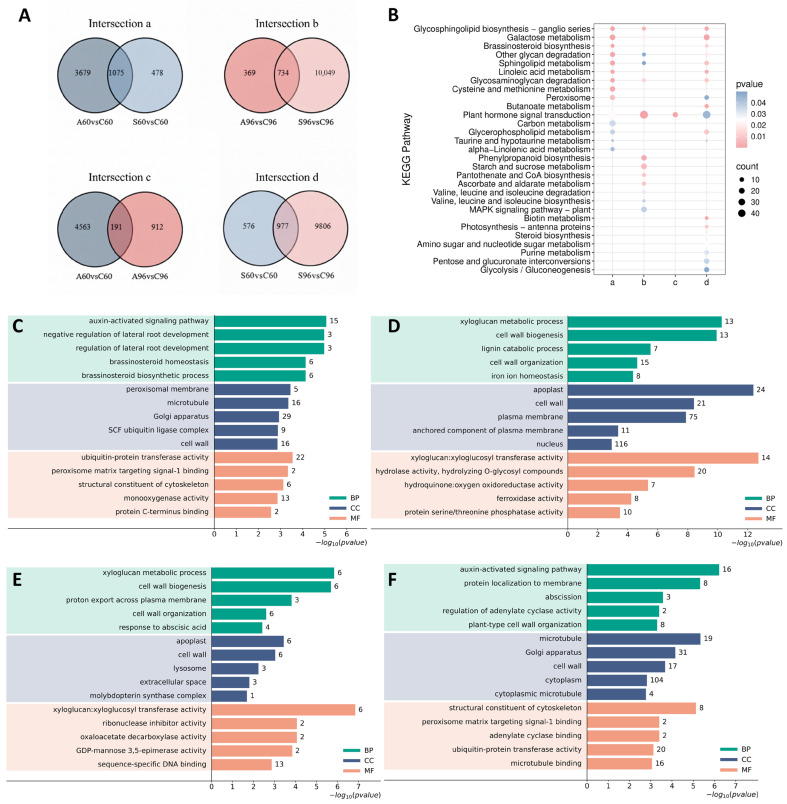
Transcriptomic overview and functional enrichment analysis of differentially expressed genes (DEGs). (**A**) Venn diagram of DEGs illustrating four defined intersection sets: a, early common response (intersection between CK60 vs. SS60 and CK60 vs. SA60); b, late common response (intersection between CK96 vs. SS96 and CK96 vs. SA96); c, alkali-specific response (intersection between CK60 vs. SA60 and CK96 vs. SA96); and d, salt-specific response (intersection between CK60 vs. SS60 and CK96 vs. SS96). (**B**) KEGG pathway enrichment analysis of the four sets. Bubble size represents the number of genes, and the color scale indicates the significance level. (**C**–**F**) GO enrichment analysis for sets a to d, respectively. Bar charts display enrichment significance and the number of enriched genes. BP: Biological Process; CC: Cellular Component; MF: Molecular Function.

Subsequent Venn diagram analysis illustrated the overlap of genes among different treatments (Figure 3A). Based on treatment duration and stress type, DEGs were categorized into four intersection sets with distinct temporal and stress-specific characteristics: The early common response set (a) contained 1075 genes, suggesting that both salt and alkali stresses activated substantial common signaling pathways during early germination; the late common response set (b) comprised 734 genes, implying that partial common responses were retained as stress persisted. Notably, significant differences were observed in continuous response patterns: the salt-specific temporal set (d) comprised 977 genes, far exceeding the 191 genes in the alkali-specific temporal set (c). This finding suggests that compared to alkali stress, CN16 maintained more extensive and persistent specific transcriptional regulation under salt stress.

KEGG pathway enrichment analysis revealed a temporal shift in metabolic regulatory priorities and treatment specificity (Figure 3B). Regarding common responses, pathways enriched in the early set (a) were primarily associated with membrane lipid remodeling and basal metabolism. Beyond galactose metabolism (ko00052), pathways integral to membrane integrity—including glycerophospholipid metabolism (ko00564), sphingolipid metabolism (ko00600), and linoleic acid metabolism (ko00591)—were significantly enriched. This reflects an early prioritization of lipid composition adjustment in CN16 to cope with osmotic shock. As stress persisted, the regulatory focus of the late set (b) shifted significantly toward reserve mobilization, secondary metabolism, and hormone signaling networks. Notably, starch and sucrose metabolism (ko00500)—central to carbon allocation—along with phenylpropanoid biosynthesis (ko00940) and plant hormone signal transduction (ko04075), emerged as core enriched pathways. This suggests that late-stage adaptation mechanisms involve the coordination of energy supply and signal regulation. Regarding specific responses, pathway enrichment in the alkali-specific set (c) was relatively restricted, predominantly involving hormone signal transduction and specific carbon metabolism processes. In contrast, the salt-specific set (d) exhibited broader metabolic regulation. Beyond basal energy pathways such as glycolysis/gluconeogenesis (ko00010), plastid-related pathways including photosynthesis–antenna proteins (ko00196) were also significantly enriched.

GO functional annotation further elucidated key cellular processes associated with distinct response patterns (Figure 3C–F). To provide a comprehensive overview, the top 10 significantly enriched GO terms for each category are detailed in Supplementary Table S2, while the top 5 are visualized in Figure 3C–F. Overall, DEGs were predominantly localized to the plasma membrane (GO:0005886) and Golgi apparatus (GO:0005794), and were extensively involved in cell wall organization or biogenesis (GO:0071555). Specifically, the functional profile of the early common set (a) was characterized by oxidative stress responses and early signal initiation. Within the response to hydrogen peroxide (GO:0042542) pathway, all 7 enriched genes were significantly up-regulated, with heat shock protein genes such as *HSP20-like* (Glyma.08G068700) being the most highly induced. Conversely, the auxin-activated signaling pathway (GO:0009734) was severely suppressed; 13 out of the 15 enriched genes exhibited down-regulation, represented most prominently by the profound repression of *AUX28-like* (Glyma.02G142500). Furthermore, the ubiquitin-protein transferase activity (GO:0004842) pathway showed dynamic protein remodeling, comprising 16 up-regulated genes (e.g., F-box/kelch-repeat protein, Glyma.15G101600) and 6 down-regulated genes (e.g., U-box domain-containing protein 30, Glyma.U029700) (Table S3a). In contrast, the late common set (b) prioritized physical barrier reinforcement and transcriptional regulation. Within the cell wall biogenesis (GO:0042546) pathway, 11 out of the 13 enriched genes were up-regulated, most notably driven by the dramatic induction of the xyloglucan endotransglucosylase/hydrolase 23-like (*XTH23-like*) gene (Glyma.13G095000). Conversely, all 8 genes enriched in iron ion homeostasis (GO:0055072) were systemically down-regulated; this profound repression was entirely represented by laccase family members, with *laccase-11* (Glyma.11G069600) being the most severely suppressed. Furthermore, massive transcriptional reprogramming was evidenced by the DNA-binding transcription factor activity (GO:0003700) term, which comprised 27 up-regulated and 16 down-regulated genes. Among these, an AP2-like DNA-binding transcription factor (Glyma.16G199000) exhibited the most extreme induction, while the transcriptional activator *PTI6* (Glyma.03G094700) showed the deepest down-regulation (Table S3b).

Notably, the specific responses to the two stress types exhibited distinct mechanistic strategies at the GO level. The alkali-specific set (c) focused intensely on maintaining acid–base homeostasis. Core enrichment in proton transmembrane transport (GO:1902600), proton export across the plasma membrane (GO:0120029), and regulation of intracellular pH (GO:0051453) demonstrated the targeted defense of CN16 against high-pH environments. Specifically, all genes enriched within these pH-regulating pathways were exclusively up-regulated, representing a dedicated, systemic activation of plasma membrane H^+^-ATPases. Among them, the induction was most severely driven by the plasma membrane ATPase 1-like (Glyma.13G065400) and plasma membrane ATPase 4 (Glyma.17G199200) genes (Table S3c). Conversely, the salt-specific set (d) centered on cytoskeleton rearrangement and lipid signaling. Significant enrichment was identified in cytoskeleton-related terms—including microtubule (GO:0005874), structural constituent of cytoskeleton (GO:0005200), which exhibited a massive and systemic collapse. For instance, 19 out of 21 enriched genes in the microtubule pathway were severely down-regulated, represented most prominently by the extreme suppression of ankyrin-3 (Glyma.16G060400) and the tubulin beta chain (Glyma.04G023900). In addition, the cellulose biosynthetic process (GO:0030244) showed dynamic remodeling with 4 up-regulated (e.g., cellulose synthase-like protein E6, Glyma.14G012800) and 3 down-regulated genes. Meanwhile, the oxylipin biosynthetic process (GO:0031408) was actively induced, with 4 out of 5 enriched genes being up-regulated, led by linoleate 13S-lipoxygenase 2-1 (Glyma.13G030300) (Table S3d). This implies that the severe arrest of cell expansion and morphology maintenance, alongside the synthesis of lipid signaling molecules, constituted the primary cellular response features uniquely triggered by neutral salt stress.

### 3.3. Weighted Gene Co-Expression Network Analysis (WGCNA) and Identification of Key Transcriptomic Hubs

**Figure 4:**
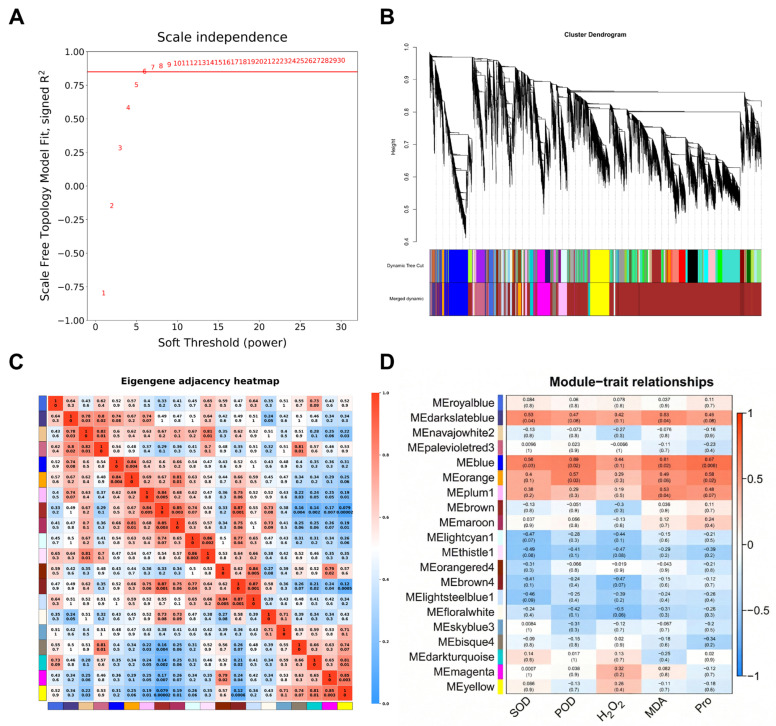
Weighted gene co-expression network analysis (WGCNA). (**A**) Analysis of the scale-free topology fit index across different soft-thresholding powers (β). The red numbers represent the tested soft-thresholding powers, and the red line indicates the threshold criterion (*R*^2^ > 0.8). (**B**) Hierarchical clustering dendrogram of genes based on topological overlap dissimilarity, identifying 20 color-coded co-expression modules. The distinct colors in the horizontal bar represent individual modules identified by the dynamic tree cut algorithm. (**C**) Eigengene adjacency heatmap visualizing correlations among module eigengenes. The color gradient from blue to red represents the correlation strength ranging from 0 to 1. (**D**) Module-trait association heatmap. Each cell displays the correlation coefficient (*r*) and the corresponding *p*-value. The color scale denotes correlation strength (red: positive correlation; blue: negative correlation).

To identify core gene co-expression networks regulating saline-alkali tolerance in soybean, a WGCNA network was constructed using transcriptomic data from all samples. A soft-thresholding power of *β* = 6 was selected as the lowest value satisfying the scale-free topology criterion (*R*^2^ > 0.8) (Figure 4A). Consequently, genes were clustered into 20 co-expression modules (Figure 4A–C). Module-trait association analysis was performed using the pre-selected core physiological trait panel (Pro, MDA, H_2_O_2_, SOD, and POD) defined in Section 2.6. The results highlighted the MEblue module as being highly associated with tolerance traits (Figure 4D). Statistical analysis revealed that MEblue was the only module significantly correlated with all five core indices simultaneously. Specifically, this module exhibited the strongest positive correlation with the osmolyte proline (Pro) (*r* = 0.67, *p* = 0.006) and significant positive correlations with MDA (*r* = 0.61, *p* = 0.02), as well as with antioxidant enzymes SOD (*r* = 0.56, *p* = 0.03) and POD (*r* = 0.59, *p* = 0.02). These specific correlations indicate that genes within the MEblue module are directly linked to stress-induced physiological defense and damage repair mechanisms.

In contrast, other modules correlated with individual indices but did not exhibit a systemic association with the entire core trait panel. For instance, the MEdarkslateblue module was significantly correlated only with MDA (*r* = 0.53, *p* = 0.04) but not with Pro (*p* = 0.09). Similarly, the MEorange module showed a significant positive correlation with Pro (*r* = 0.58, *p* = 0.02) but no significant association with MDA (*p* = 0.06). Given that the MEblue module statistically captured key physiological features of both osmotic adjustment and antioxidant defense, it was designated as the core hub module for dissecting mechanisms underlying saline-alkali tolerance.

To pinpoint the most critical genes within this hub module, a two-step screening method was applied based on network connectivity and biological responsiveness. First, genes with a high module membership (kME ≥ 0.90) were selected to ensure they represent the core co-expression pattern of the network. Second, these topological hubs were cross-referenced with the differentially expressed gene (DEG) sets (a–d) to confirm their significant response to saline-alkali stress.

Ultimately, five key candidate genes were identified. According to their functional annotations, these five candidate genes were classified into two main biological categories: regulatory genes involved in signaling, and genes encoding metabolic or structural proteins. From the early common response set (a), two regulatory genes were identified: *GmPHOT2b* (Glyma.16G096600), a member of the AGC kinase family typically implicated in environmental sensing and intracellular signal transduction; and the cytokinin-activating enzyme *GmLOG* (Glyma.12G174900), which is involved in hormone-mediated stress responses (notably, also present in the salt-specific set (d)). The remaining three genes encode proteins primarily related to basal metabolism and cellular protection, potentially serving as downstream effectors. The alkali-specific set (c) included *GmSHMT08* (Glyma.08G274400), annotated as a serine hydroxymethyltransferase. This enzyme acts as a core node in one-carbon metabolism, a pathway known to supply essential intermediates and reducing equivalents for cellular homeostasis. The late common response set (b) yielded Glyma.01G121700, which harbors a rhodanese-like domain generally associated with sulfur metabolism and detoxification processes, alongside cell cycle regulation-related domains. Additionally, Glyma.08G320400 (from set (a)) was identified as a putative stress-responsive structural gene. 

### 3.4. Untargeted Metabolomics Analysis and Identification of Differentially Accumulated Metabolites (DAMs)

Untargeted metabolomic profiling was performed to investigate the metabolic responses of the tolerant cultivar CN16 to neutral and alkaline salt stresses during germination. Principal component analysis (PCA) demonstrated that PC1 (34.2%) primarily captured variance associated with germination time, effectively separating the 60 h and 96 h samples (Supplementary Figure S1). In parallel, PC2 (17.5%) significantly differentiated between treatments; CK, SS, and SA samples exhibited a distinct gradient distribution, demonstrating high intra-group reproducibility.

Consistent with the PCA results, the identification of differentially accumulated metabolites (DAMs) indicated that both stresses triggered extensive and sustained metabolic reprogramming. The comprehensive dataset and quantitative profiles of all identified DAMs across the four comparison groups are provided in Supplementary Table S4. At 60 h, SA treatment elicited 3676 DAMs (1828 upregulated and 1848 downregulated), a magnitude comparable to that of SS treatment, which yielded 3690 DAMs (2062 upregulated and 1628 downregulated). By 96 h, the number of DAMs under SA and SS treatments remained high at 3353 and 3472, respectively, with both conditions characterized by a bias toward upregulated metabolites (Table 2). These findings imply that the metabolic response to SA was rapidly activated during early germination, whereas the response to SS was sustained at a high intensity throughout the stress period, predominantly favoring metabolite accumulation.

**Figure 5:**
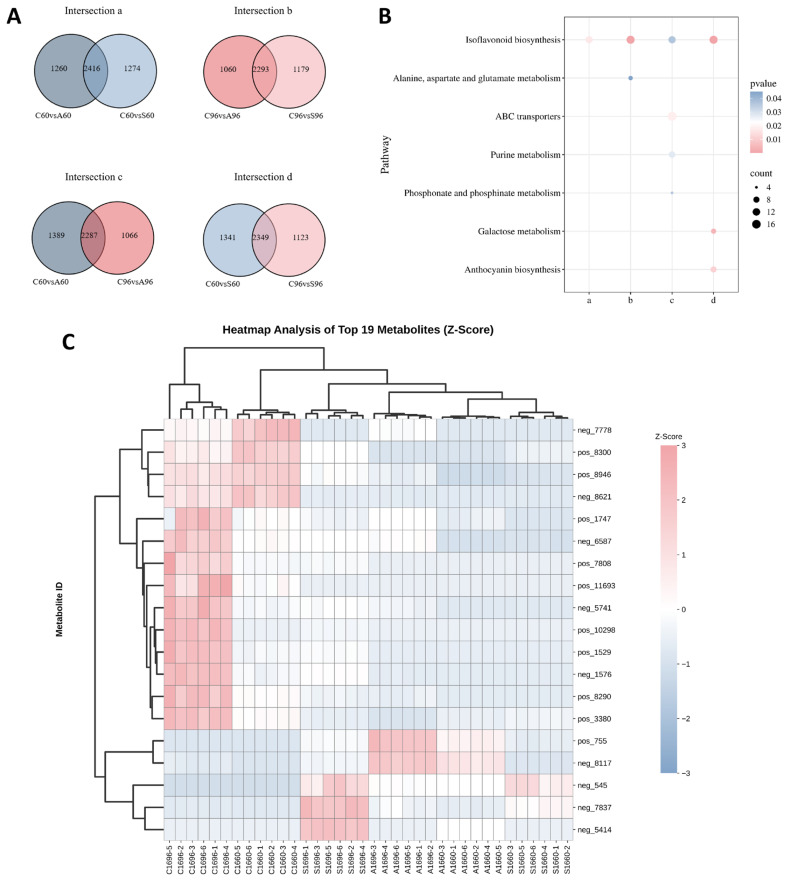
Overview of untargeted metabolomics analysis and identification of key metabolites. (**A**) Venn diagrams depicting the four intersection sets (a–d) of differentially accumulated metabolites (DAMs). Intersections a and b represent the overlap between neutral and alkaline salt responses at 60 h and 96 h, respectively. Intersections c and d represent the temporal overlap (60 h vs. 96 h) under alkaline and neutral salt treatments, respectively. (**B**) KEGG pathway enrichment results for the four intersection sets. (**C**) Bidirectional hierarchical clustering heatmap of the 19 identified core metabolites (Z-score).

To elucidate common and specific metabolic characteristics under neutral and alkaline salt stresses, DAMs were screened based on strict criteria (VIP ≥ 1, *p* < 0.05, and |log_2_FC| ≥ 1) and categorized into four intersection sets (Figure 5A).

Focusing on the early (set a) and late (set b) common response sets, chemical classification analysis (Supplementary Figure S2) unveiled similar metabolic profiles, predominantly characterized by lipids and lipid-like molecules as well as phenylpropanoids and polyketides. This chemical profile mirrored the KEGG enrichment results (Figure 5B): isoflavonoid biosynthesis (ko00943) was significantly enriched in both sets. Additionally, set b exhibited significant enrichment in alanine, aspartate, and glutamate metabolism (ko00250).

In the alkali-specific set (c), chemical classification pinpointed carboxylic acids and derivatives as the predominant category, comprising the highest proportion (Supplementary Figure S2). Corroborating the abundance of organic acids, KEGG analysis revealed significant enrichment in ABC transporters (ko02010)—implicated in transmembrane transport—as well as in purine metabolism (ko00230) and phosphonate and phosphinate metabolism (ko00440).

For the salt-specific set (d) (Supplementary Figure S2), chemical profiling illustrated that the proportions of phenylpropanoids, polyketides, and organooxygen compounds were relatively prominent, whereas carboxylic acids were substantially diminished. KEGG functional annotation indicated (Figure 5B) that this set was simultaneously enriched in anthocyanin biosynthesis (ko00942) and galactose metabolism (ko00052).

Based on the multi-stage, stringent screening strategy defined in Section 2.5.3, we sought to distill the most robust metabolic effectors from the highly dimensional datasets. A Z-score heatmap was generated using the top 20 DAMs ranked by maximum absolute $\left|\log_{2}\mathrm{Foldchange}\right|$ from each intersection set (Supplementary Figure S3) to isolate features with the most profound biological effect sizes. Following the strict exclusion of any candidates with incomplete quantitative data across biological replicates, a final, highly confident panel of 19 core metabolites was retained for bidirectional hierarchical clustering analysis (Figure 5C). Notably, four metabolites—7*Z*-octadecen-11-one (pos10298), *R*-19-methyl-1-eicosyn-3-ol (pos11693), norepinephrine sulfate (pos1529), and delphinidin 3-*O*-sophoroside (pos755)—were detected across all four intersection sets.

The Z-score heatmap (Supplementary Figure S3) revealed distinct metabolic response patterns across the different sets. In the early common response set (a), metabolite responses exhibited a divergent trend: the indole derivative 7-hydroxy-2-oxoindole-3-acetic acid 7′-*O*-glucopyranoside (neg1554) accumulated significantly under SA stress (Z-score: 1.41 vs. CK: −0.76), whereas the flavonoid 3-hydroxyflavone (pos8946) was significantly suppressed (CK: 1.41 vs. SA: −0.80). The late common response set (b) was characterized primarily by stress-induced suppression: the lupin alkaloid (+)-13β-hydroxymamanine (pos8360) and the non-protein amino acid (*S*)-hypoglycin A (neg1576) maintained high abundance in the control group (Z-score > 1.3) but were sharply downregulated under SA stress (Z-score < −0.7).In the alkali-specific set (c), the anthocyanin delphinidin 3-*O*-sophoroside (pos755) displayed strong specific induction (SA Z-score: 1.55; other groups < −0.8). Conversely, in the salt-specific set (d), the alkaloid procerine (neg5742) was specifically highly accumulated under SS treatment (Z-score: 1.73 vs. CK: −0.58).

Bidirectional hierarchical clustering analysis of the 19 core metabolites (Figure 5C) revealed that the sample dendrogram primarily distinguished the control group from the stress treatment groups. Within the stress clade, 60 h samples clustered tightly due to similar metabolic profiles, whereas 96 h samples displayed distinct separation between treatments.

Metabolite clustering identified two clusters with distinct abundance patterns: The stress-induced cluster was represented by the anthocyanin delphinidin 3-*O*-sophoroside (pos755), the oxylipin 12,13-epoxy-9-hydroxy-10-octadecenoate (neg7837), and the monoglyceride 1-monostearin (neg8117). These metabolites exhibited negative Z-scores in the CK group but shifted significantly to positive values under stress, demonstrating typical stress-inducible characteristics. Conversely, the stress-suppressed cluster was typified by the nitrogen-containing compounds norepinephrine sulfate (pos1529) and (S)-hypoglycin A (neg1576), the isoflavone lupinisoflavone A (pos8300), and the aliphatic ketone 7*Z*-octadecen-11-one (pos10298). These metabolites maintained high Z-scores in the CK group but were rapidly downregulated to negative values under SS and SA treatments.

The dynamic remodeling of these core metabolites, combined with transcriptomic data, provides a robust molecular evidence chain. Specifically, the accumulation of anthocyanins concomitant with the reduction in isoflavones aligns precisely with the transcriptional activation of phenylpropanoid biosynthesis (ko00940) observed in the late response set. Furthermore, the significant accumulation of membrane lipid components—such as oxidized fatty acids and monoglycerides—strongly corroborates the enrichment of glycerophospholipid metabolism (ko00564) and linoleic acid metabolism (ko00591) in the early response set, as well as the upregulation of the oxylipin biosynthetic process (GO:0031408) in the salt-specific set. Collectively, these results suggest that modulating flux through the phenylpropanoid branch to enhance antioxidant capacity, alongside remodeling membrane lipid composition to maintain membrane integrity, represents a core metabolic strategy employed by soybean seeds to cope with saline-alkali stress.

### 3.5. Integrated Analysis of Transcriptome and Metabolome

**Figure 6:**
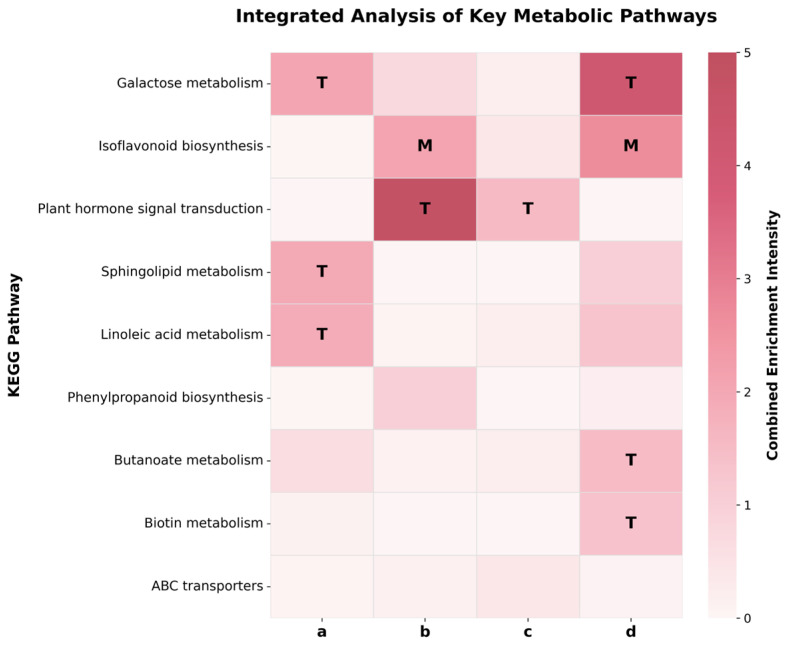
Heatmap of integrated KEGG pathway enrichment analysis. The heatmap visualizes the combined enrichment intensity of nine key KEGG metabolic pathways across four intersection sets: (a) early common response, (b) late common response, (c) alkali-specific response, and (d) salt-specific response. The color gradient reflects the aggregate significance rank, derived from the integration of transcriptomic and metabolomic FDR values. Superscript annotations denote the primary driving factor of enrichment: ‘T’ indicates transcriptome-driven enrichment (*Q*_DEG_ < 0.05); ‘M’ indicates metabolome-driven enrichment (*Q*_DAM_ < 0.05).

To elucidate the synergistic characteristics of gene expression and metabolite accumulation, DEGs and DAMs were mapped to shared metabolic pathways using the KEGG database. The combined enrichment intensity of each pathway was evaluated to prioritize key regulatory nodes at both transcriptomic and metabolomic levels (Figure 6). The detailed source data and calculated integration strength scores for all mapped pathways are provided in Supplementary Table S5.

Integrated analysis of the early common response set (a) highlighted three top-ranking pathways: galactose metabolism (ko00052), sphingolipid metabolism (ko00600), and linoleic acid metabolism (ko00591). Activity in these pathways was driven exclusively by substantial changes at the transcriptomic level, whereas metabolite abundance remained non-significant (*Q*_DAM_ > 0.05). This observed lag between transcriptional activation and metabolic accumulation indicates that the early stress response (60 h) was characterized primarily by the upregulation of relevant enzyme-coding genes, preceding the stable accumulation of metabolites.

As stress extended to 96 h, soybean metabolic priorities shifted significantly. The late common response set (b) identified isoflavonoid biosynthesis (ko00943) and phenylpropanoid biosynthesis (ko00940) as core pathways. Enrichment of the isoflavonoid pathway was driven primarily by metabolomic changes (*Q*_DAM_ = 0.008), characterized by a significant reduction in isoflavonoids such as lupinisoflavone A. In contrast, the upstream phenylpropanoid biosynthesis pathway maintained marginally significant enrichment at the transcriptomic level (*Q*_DEG_ = 0.096). The discrepancy between sustained upstream gene expression and reduced downstream metabolite abundance highlights a shift in metabolic flux within the phenylpropanoid pathway. Furthermore, plant hormone signal transduction (ko04075) exhibited highly significant transcriptomic enrichment (*Q*_DEG_ < 0.0001), underscoring the integral role of hormone signaling networks in late-stage metabolic regulation. Although few pathways achieved stringent statistical significance (Q < 0.05) simultaneously in both omics layers at a single time point, this overall discrepancy highlights the inherent biological time lag between transcriptomic activation and subsequent metabolite accumulation or depletion.

In the alkali-specific set (c), the molecular profile centered on intracellular buffering processes. Transcriptomic GO analysis highlighted terms such as proton transmembrane transport (GO:1902600), regulation of intracellular pH (GO:0051453), and proton export across the plasma membrane (GO:0120029). At the KEGG level, plant hormone signal transduction (ko04075) was the sole pathway reaching significant enrichment (QDEG = 0.028). Notably, integration with metabolomic data highlighted ABC transporters (ko02010) as well as ascorbate and aldarate metabolism (ko00053). While their transcriptomic enrichment was moderate, these pathways aligned with the metabolomic profile, where carboxylic acids and derivatives constituted the most abundant chemical category. Differential metabolite analysis further demonstrated the specific accumulation of the anthocyanin delphinidin 3-O-sophoroside under SA treatment, suggesting a coordinated molecular signature involving organic acid transport and anthocyanin synthesis.

The salt-specific set (d) was characterized by two high-ranking core pathways with distinct driving mechanisms. Galactose metabolism (ko00052) was identified as a key pathway driven solely by substantial changes at the transcriptomic level (*Q*_DEG_ = 0.0005), involving the upregulation of genes responsible for galactose and oligosaccharide synthesis. Conversely, isoflavonoid biosynthesis (ko00943) exhibited a metabolome-driven pattern (*Q*_DAM_ = 0.006). Coupled with the high proportion of phenylpropanoids and polyketides observed in the chemical classification, this significant shift in metabolite abundance signifies a rapid turnover of the secondary metabolite pool. Furthermore, butanoate metabolism (ko00650) and biotin metabolism (ko00780) also reached significant enrichment levels at the transcriptomic level (*Q*_DEG_ < 0.05), suggesting a synchronous enhancement of stress signaling and basal metabolic cofactors under salt stress.

To further resolve the direct regulatory couplings that are often obscured by the biological time lag in canonical KEGG pathway mapping, a targeted Pearson correlation analysis was performed. We evaluated the statistical co-variation between the FPKM values of the 5 critical transcriptomic hub genes and the relative abundances of the 19 core stress-responsive metabolites across all treatments.

**Figure 7:**
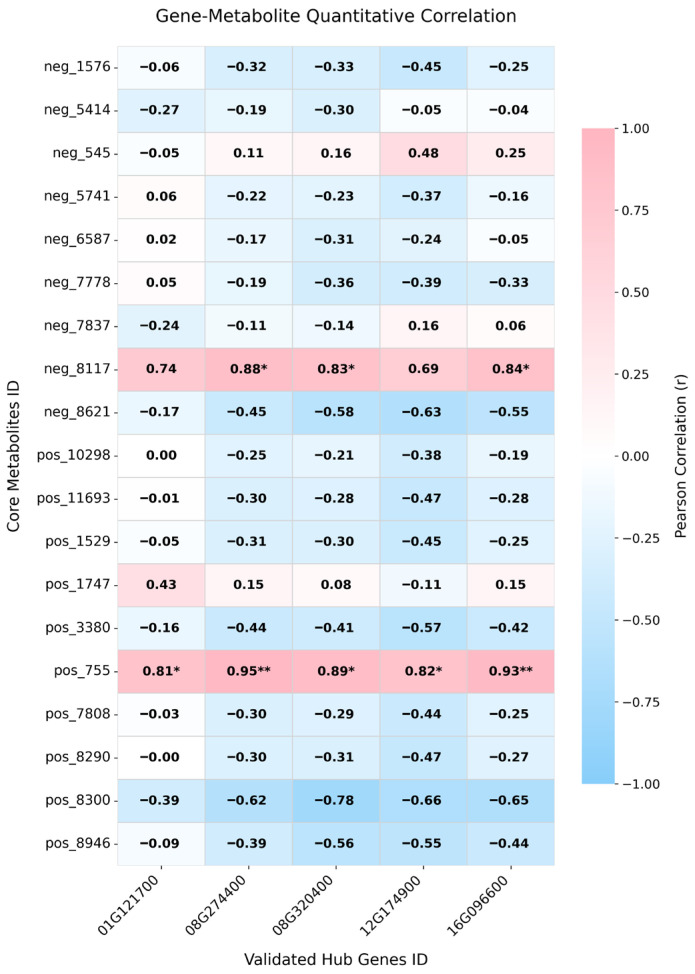
Targeted quantitative correlation analysis between transcriptomic hub genes and core stress-responsive metabolites. The heatmap visualizes the Pearson correlation coefficients (*r*) between the FPKM values of the five WGCNA-derived hub genes (*x*-axis) and the relative abundances of the 19 core metabolites (y-axis) across all saline-alkali stress and control treatments (60 h and 96 h). The color gradient from deep blue to deep red represents negative (*r* < 0) to positive (*r* > 0) correlations, respectively. Statistical significance of the correlation is denoted by asterisks: * indicates *p* < 0.05; ** indicates *p* < 0.01.

The resulting correlation heatmap (Figure 7) detailed the Pearson correlation coefficients (*r*) for these gene-metabolite pairs. Based on the stringent criteria of *r* > 0.8 and *p* < 0.05, significantly strong positive correlations were selectively restricted to only two metabolic effectors. Specifically, the anthocyanin delphinidin 3-O-sophoroside (pos_755) exhibited significant positive correlations with all five hub genes (*r* ranging from 0.81 to 0.95), with *GmSHMT08* (*r* = 0.95, *p* < 0.01) and *GmPHOT2b* (*r* = 0.93, *p* < 0.01) demonstrating the strongest statistical coupling. Similarly, the membrane lipid 1-monostearin (neg_8117) was significantly and positively correlated with three of the hub genes (*r* ranging from 0.83 to 0.88, *p* < 0.05). The absence of strong correlations (*r* < 0.8 or *p* > 0.05) with the remaining 17 core metabolites indicates that the transcriptional changes in this 5-gene module are specifically coupled with the accumulation of delphinidin 3-O-sophoroside and 1-monostearin, rather than reflecting a general up-regulation of the secondary metabolite pool.

### 3.6. Functional Validation of Core Regulatory Hubs via qRT-PCR

Based on the targeted correlation analysis, the five WGCNA-derived hub genes were shown to tightly coordinate with the accumulation of critical stress-responsive metabolites, particularly the antioxidant delphinidin 3-O-sophoroside and the membrane lipid 1-monostearin. To confirm the functional relevance of this specific regulatory module, these exact five hub genes were selected for qRT-PCR validation, avoiding the bias of random DEG sampling.

**Figure 8:**
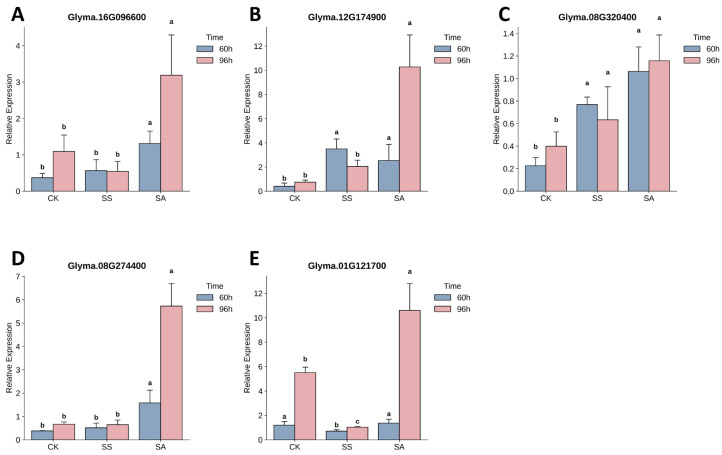
Expression validation of the five network-derived core regulatory hub genes under neutral and alkaline salt stresses. (**A**) *GmPHOT2b* (Glyma.16G096600). (**B**) *GmLOG* (Glyma.12G174900). (**C**) Glyma.08G320400. (**D**) *GmSHMT08* (Glyma.08G274400). (**E**) Glyma.01G121700. Relative expression levels of the targeted hubs were quantified using qRT-PCR across control (CK), neutral salt (SS), and alkaline salt (SA) treatments at 60 h and 96 h. Data represent the mean ± standard deviation (SD) of three independent biological replicates. Different lowercase letters above the bars indicate statistically significant differences among treatments within the same time point at *p* < 0.05, according to Duncan’s multiple range test.

The expression patterns of these candidate genes were analyzed across all treatments and time points (primers listed in Supplementary Table S6). As shown in Figure 8, the transcript levels of all five genes were significantly induced by SA treatment compared to the CK and SS groups. For instance, the expression of Glyma.01G121700 reached its peak abundance at 96 h under SA stress. Overall, the expression trends captured by qRT-PCR closely matched the RNA-seq data, thereby validating the reliability of the transcriptomic sequencing (Supplementary Figure S4) and confirming the robust activation of these core genes under alkaline salt stress.

## 4. Discussion

### 4.1. Physiological Barriers and Metabolic Responses of Soybean Seed Germination Under Saline-Alkali Stress

This study confirmed that under equimolar Na^+^ conditions, alkaline salt stress caused significantly greater inhibition of germination and hypocotyl elongation across all genotypes compared to neutral salt (SS) stress, accompanied by severe radicle browning and necrosis. This differential growth suppression is primarily attributable to the multiple physiological limits caused by the high-pH environment. Beyond the shared mechanisms of ion toxicity and osmotic stress, alkali stress triggered a stronger oxidative burst, leading to severe peroxidative damage to membrane systems, as evidenced by significantly elevated levels of hydrogen peroxide (H_2_O_2_) and superoxide anions ($O_{2}^{-}$), coupled with sharp increases in MDA content and relative electrical conductivity (REC) under alkaline salt stress [6]. Furthermore, high extracellular pH not only reduced the bioavailability of key mineral nutrients (e.g., Fe, Zn, and P) [35,36] but also inhibited the proton secretion process required for cell wall acidification by negatively regulating plasma membrane H^+^-ATPase activity, thereby directly physically restricting hypocotyl elongation [37].

While tolerant cultivars retained relatively high germination rates under alkaline salt stress, biomass accumulation was significantly reduced. From a bioenergetic perspective, this growth retardation represents an unavoidable metabolic result of the high energy consumption required to mitigate intracellular ion toxicity and buffer extreme alkaline stress [38]. Established physiological models indicate that under alkaline conditions, plants are required to intensively upregulate specific ion transporters and synthesize substantial amounts of organic acids to counteract the acid-base changes induced by extracellular alkalinity [10,39]. Given that establishing transmembrane ion gradients and reprogramming metabolic flux are processes with high energy demand [40], the suppression of hypocotyl elongation under alkaline salt stress inherently reflects a redistribution of energy resources (e.g., ATP) from cell division and elongation toward survival-oriented stress buffering mechanisms.

Under these stress conditions, the difference in tolerance between genotypes is determined mainly by the stability of antioxidant systems and the capacity for osmotic adjustment. In sensitive cultivars, antioxidant enzyme activities decreased significantly, and MDA content increased sharply during the late stress stage, indicating a loss of membrane system integrity due to severe damage. In contrast, tolerant cultivars effectively reduced oxidative damage and maintained cell turgor pressure by sustaining high levels of SOD, POD, and catalase (CAT) activities, as well as accumulating proline. These physiological responses are consistent with the typical traits of alkali-tolerant plants, which activate ROS signaling and metabolic defense networks [41]. This capability allows the plants to retain sufficient energy for survival and basic growth, despite the large energy consumption required for metabolic buffering and ion compartmentalization. Because CN16 showed the strongest tolerance, we selected it as the primary material to analyze the molecular mechanisms of saline-alkali tolerance in soybean.

### 4.2. Multi-Omics Integration Reveals Stage-Specific Adaptation and Metabolic Flux Redirection

This study utilized an integrated transcriptomic and metabolomic approach to dissect the temporal dynamics of gene expression and metabolite accumulation, uncovering the stage-specific regulatory strategies of soybean seeds during saline-alkali germination [42].

At the early stress stage, our integrated analysis (Set a) revealed that responses in core pathways—such as galactose, sphingolipid, and linoleic acid metabolism—were driven exclusively at the transcriptional level, while the corresponding terminal metabolites did not exhibit significant accumulation. Cao et al. [43] demonstrated that during the initial phase of salt shock, plants rapidly upregulate genes for cell wall and membrane lipid remodeling to mitigate structural damage. Our 60 h transcriptomic data perfectly align with this early structural defense priming. It is highly probable that the intermediate lipid and carbohydrate molecules synthesized during this early phase undergo rapid turnover—being immediately consumed for membrane repair rather than accumulating as stable end products.

As stress extended to 96 h, the multi-omics data (Set b) provided direct molecular evidence for a “growth–defense trade-off”. Deinlein et al. [44] established that salt tolerance requires plants to actively restrict basic growth metabolism to conserve resources for defense. In our phenylpropanoid and isoflavonoid biosynthesis pathways, a clear omics divergence emerged: while upstream regulatory genes maintained active expression, the downstream metabolome exhibited a severe depletion of growth-promoting isoflavonoids (e.g., lupinisoflavone A). Concurrently, stress-responsive secondary metabolites, particularly the potent antioxidant delphinidin 3-O-sophoroside, accumulated significantly. This dynamic shift confirms that finite carbon skeletons are selectively diverted away from secondary growth branches toward the synthesis of terminal ROS-scavenging effectors. Ensuring the functionality of these defense metabolites without causing cytosolic toxicity requires active tonoplast transport [45], a process confirming the strict reallocation of carbon resources toward subcellular defense.

Crucially, the multifold greater inhibition of germination under alkaline stress compared to neutral salt stress can be directly explained by their distinct metabolic response profiles. For neutral salt stress, the specific response (Set d) was characterized by a transcriptome-driven enhancement of galactose metabolism, indicating that soybeans primarily deploy soluble sugars for classical osmotic adjustment.

In stark contrast, mitigating alkaline stress imposes a substantially broader metabolic reconfiguration. Studies by Zhou et al. [46] and Li et al. [47] demonstrated that plants counter severe external alkalinity by activating plasma membrane H^+^-ATPases to excrete protons and synthesizing large quantities of organic acids to buffer cellular toxicity. Our alkali-specific omics data (Set c) provides direct factual support for this mechanism: we observed the systematic transcriptomic upregulation of proton transmembrane transport genes, coupled with a massive metabolomic accumulation of carboxylic acids. Furthermore, the highly specific enrichment of ABC transporters (ko02010) under SA treatment highlights the necessity of actively compartmentalizing these massive organic anions into the vacuole. Shabala et al. [48] noted that the operation of such active transport machinery and the maintenance of transmembrane ion gradients require continuous ATP hydrolysis. Therefore, the continuous pumping of massive organic anions into the vacuole under alkali stress directly consumes the energy reserves that would otherwise fuel cell division and radicle elongation, providing a concrete biochemical explanation for the severe germination arrest.

### 4.3. Molecular Regulation of Adaptation Mechanisms by Key Candidate Genes

The profound metabolic reconfiguration and severe germination arrest discussed above do not merely reflect passive stress damage; rather, they represent a highly regulated physiological response. Initiating this systemic shift from primary growth to antioxidant defense requires specific upstream genes to perceive environmental cues and activate the corresponding biochemical pathways. To identify these core genetic regulators, we focused on the WGCNA-derived MEblue module and its gene–metabolite correlation patterns. The results suggest that these hub genes are organized into a coordinated network linking stress perception, hormonal regulation, redox homeostasis, and membrane-associated adjustment. Among the detected metabolites, the antioxidant delphinidin 3-O-sophoroside (pos755) showed significant positive correlations with all five hub genes. Meanwhile, 1-monostearin (neg8117), a lipid remodeling intermediate, showed significant positive correlations with three specific genes (*GmPHOT2b*, *GmSHMT08*, and Glyma.08G320400). These patterns indicate that anthocyanin accumulation and lipid-related metabolic adjustment represent two linked, but differentially regulated, outputs of this central regulatory network.

Within this module, the blue-light receptor *GmPHOT2b* (Glyma.16G096600) and the cytokinin-activating enzyme *GmLOG* (Glyma.12G174900) exhibited a strong co-expression pattern, suggesting that early signal perception and hormonal regulation may act in concert. Although photoreceptors are classically associated with aerial tissues, Liu et al. [49] reported that the promoter region of *GmPHOT2b* is enriched in salt stress- and ABA-responsive elements, implying that this gene may also function as a stress sensor in root tissues. In CN16, the induction of *GmPHOT2b* may therefore represent an early signaling event that helps the seed perceive saline-alkali stress and initiate downstream responses. Subsequently, *GmLOG* may contribute to cytokinin activation and support root meristem activity under osmotic stress [50], which is consistent with the relatively better radicle growth observed in CN16. Importantly, both genes were positively correlated with delphinidin 3-O-sophoroside (pos755), suggesting that this sensory–hormonal axis is associated with the activation of antioxidant metabolism. In addition, *GmPHOT2b* also showed a significant positive correlation with 1-monostearin (neg8117), indicating that early stress perception may simultaneously participate in signaling lipid-related adjustments.

The alkali-responsive gene *GmSHMT08* (Glyma.08G274400) provides further evidence for metabolic adjustment during stress adaptation. It encodes a serine hydroxymethyltransferase involved in one-carbon metabolism, a pathway that provides essential methyl donors and contributes to cellular redox balance [51]. Previous work in rice by Mishra et al. [52] showed that overexpression of SHMT homologs can reduce salt-induced ROS accumulation, supporting a role for this gene family in oxidative stress mitigation. In CN16, *GmSHMT08* showed a strong positive correlation with delphinidin 3-O-sophoroside (pos755) and a significant positive correlation with 1-monostearin (neg8117). This pattern suggests that *GmSHMT08* may participate in supplying precursors for both antioxidant synthesis and lipid-associated metabolic adjustments. Rather than acting as a single downstream effector, *GmSHMT08* may help maintain redox homeostasis while supporting the necessary metabolic conditions for stress adaptation.

For Glyma.01G121700, identified in the late common response set, phylogenetic analysis by Landis et al. [53] classified it as a conserved gene containing a rhodanese-like domain. Based on its established role as a sulfur carrier [54], we propose that this gene may contribute to CN16 tolerance through redox maintenance and metabolic recovery during the later phase of the stress response. Rhodanese-related proteins are associated with sulfane sulfur-mediated antioxidant activity [55] and the repair of iron-sulfur (Fe-S) cluster prosthetic groups [56]. This is consistent with the enrichment of iron ion homeostasis pathways in the late transcriptomic set. Furthermore, its significant positive correlation with delphinidin 3-O-sophoroside (pos755) suggests that Glyma.01G121700 supports the protective antioxidant state. Therefore, CN16 may utilize this pathway not only to limit ROS damage but also to preserve or restore damaged metabolic components.

Taken together, the integrated data demonstrate that CN16 responds to saline-alkali stress through a coordinated regulatory network rather than a single protective pathway. Delphinidin 3-O-sophoroside (pos755) appears to represent a major shared downstream output of the hub-gene module, likely associated with enhanced antioxidant capacity and ROS mitigation. In contrast, 1-monostearin (neg8117) reflects a second branch of the response involving lipid remodeling and membrane-associated adjustment. The hub genes may therefore contribute to this adaptation at different levels: *GmPHOT2b* and *GmLOG* participate in early stress sensing and hormonal regulation; *GmSHMT08* and Glyma.01G121700 support metabolic precursor supply and enzymatic maintenance; and other components, such as Glyma.08G320400, act in concert to channel stress cues into protective metabolic adjustments. This integrated response provides an objective explanation for the superior saline-alkali tolerance of CN16 while remaining strictly consistent with the correlation data and functional evidence.

### 4.4. Phase-Specific Responses and Energy-Growth Trade-Offs Under Saline-Alkali Stress

**Figure 9:**
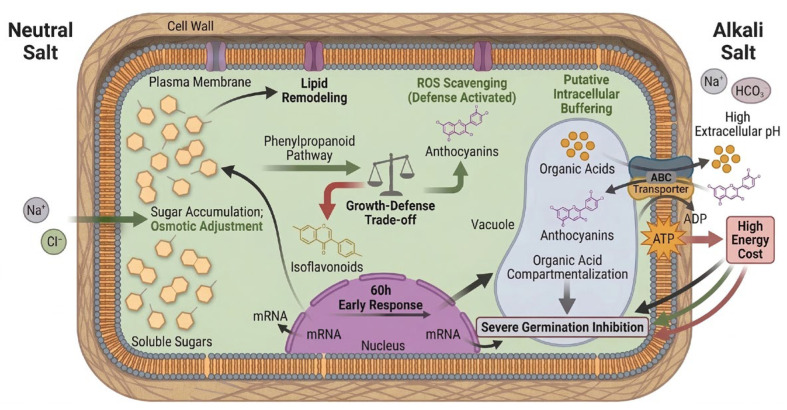
Schematic representation of the temporal response and energy-growth trade-off strategies in soybean during saline-alkali germination. The diagram illustrates two distinct phases of response: the early phase (60 h), characterized by transcriptional priming for the reinforcement of physical barriers, including cell walls and plasma membranes; and the late phase, highlighting a growth-defense trade-off involving the redirection of metabolic flux within the phenylpropanoid pathway from growth-related isoflavonoids to defense-related anthocyanins for ROS scavenging. In contrast to the relatively low-energy osmotic adjustment process under neutral salt stress, alkaline salt stress likely induces an energy-intensive intracellular buffering process. Supported by our omics data, this putative process requires the active vacuolar compartmentalization of organic acids and anthocyanins via ABC transporters. The substantial ATP consumption required for this transport process reduces the energy resources available for basal growth, ultimately leading to the severe germination inhibition observed under high-pH conditions. Note: Green and red arrows indicate activation/promotion and inhibition/suppression, respectively; thick colored arrows represent the predominant direction of metabolic flux. Black arrows indicate the causal progression of cellular events, linking early transcriptional responses to downstream metabolic adaptations and ultimate phenotypic outcomes.

By integrating the macroscopic metabolic shifts with the upstream genetic regulatory network identified above, we outline the temporal and energetic dynamics of soybean seed adaptation during saline-alkali germination (Figure 9). This integrated perspective not only defines two distinct temporal phases of response but also fundamentally differentiates the metabolic pathways and physiological limits under neutral versus alkaline salt stress.

Temporally, the adaptation follows a highly coordinated sequence. In the early phase (60 h), the system exhibits transcriptional priming, specifically upregulating genes encoding enzymes for cell wall reinforcement and lipid remodeling to construct initial physical barriers. Subsequently, in the late phase, a systemic redirection of metabolic flux occurs within the phenylpropanoid pathway. Carbon flow shifts from the growth-related isoflavonoid branch (repression) to the defense-related anthocyanin branch (activation) for ROS scavenging. While this resource reallocation structurally restricts hypocotyl elongation, it maximizes the antioxidant capacity required for survival. The five core genes discussed previously act to coordinate these stage-specific responses, seamlessly linking early stress perception to downstream chemical and physical defense outputs.

Crucially, regarding specific stress types, the integrated data highlight a fundamental divergence in energy requirements. Under neutral salt stress, the adaptive response relies primarily on osmotic adjustment, a process driven by the accumulation of soluble sugars. In stark contrast, the omics profiles under alkaline salt stress strongly imply the activation of an energy-intensive intracellular buffering process. Although intracellular pH was not directly quantified in this study, the substantial accumulation of organic acids and the specific enrichment of ABC transporters suggest a high physiological demand for the active vacuolar compartmentalization of organic acids and anthocyanins. As depicted in Figure 9, this putative transport process consumes ATP, resulting in a high energy cost. This continuous energy expenditure directly depletes the carbon and ATP pools available for basal metabolism, thereby providing a definitive biochemical explanation for the severe germination arrest observed specifically under high-pH alkaline stress.

## 5. Conclusions

This study confirms that the significantly stronger inhibition of soybean germination under alkaline salt stress compared to neutral salt stress is fundamentally driven by distinct metabolic energy requirements. While the response to neutral salt stress depends on relatively low-energy osmotic adjustment via soluble sugar accumulation, the adaptation to the high-pH environment of alkaline salt stress requires energy-intensive processes, specifically the active vacuolar compartmentalization of organic acids and anthocyanins for intracellular buffering. This substantial consumption of ATP directly reduces the energy and carbon resources available for biomass accumulation, thereby explaining the severe growth retardation observed under alkaline conditions. Furthermore, multi-omics analysis reveals a staged response pattern in the tolerant cultivar CN16, characterized by transcriptional priming for physical barrier reinforcement and lipid remodeling during the early stage, and a redirection of metabolic flux from growth-related isoflavonoids to defense-related anthocyanins during the late stage. The identification of a core regulatory module driven by key genes such as *GmPHOT2b*, *GmLOG*, and *GmSHMT08* provides the genetic basis supporting this coordination between signal detection, morphological remodeling, and metabolic detoxification. Collectively, these findings elucidate that saline-alkali tolerance depends on the regulated management of energy resources, providing specific genetic targets for breeding varieties that balance stress survival with growth potential.

## Figures and Tables

**Table 1:** Summary of differentially expressed genes (DEGs) under different comparisons.

Group	DEGs Total	DEGs Up	DEGs Down
CK60 vs. SA60	4754	2386	2368
CK60 vs. SS60	1553	736	817
CK96 vs. SA96	1103	727	376
CK96 vs. SS96	10,783	4786	5997

**Table 2:** Summary of differentially accumulated metabolites (DAMs) under different comparisons.

Group	DAMs Total	DAMs Up	DAMs Down
CK60 vs. SA60	3676	1828	1848
CK60 vs. SS60	3690	2062	1628
CK96 vs. SA96	3353	1852	1501
CK96 vs. SS96	3472	1856	1616

## Data Availability

The RNA-seq datasets generated during the current study are available in the NCBI Sequence Read Archive (SRA) repository under BioProject accession number PRJNA1421044. The metabolomics data and all other supporting results are included within the article and its Supplementary Materials.

## Supplementary Materials

The following supporting information can be downloaded at: https://www.mdpi.com/article/10.3390/biology15090670/s1, Figure S1: Principal component analysis (PCA) score plot illustrating sample distribution; Figure S2: Chemical classification of differentially accumulated metabolites (DAMs) within the four intersection sets; Figure S3: Z-score heatmap of the top 20 DAMs from each intersection set; Figure S4: Linear regression analysis of the correlation between qRT-PCR and RNA-seq data; Table S1: Summary of transcriptome sequencing quality control and the detailed list of differentially expressed genes (DEGs); Table S2: Top 10 significantly enriched gene ontology (GO) terms for each response set; Table S3: Key differentially expressed genes (DEGs) associated with the enriched GO terms across the four intersection sets; Table S4: Comprehensive dataset and quantitative profiles of differentially accumulated metabolites (DAMs) across the four comparison groups; Table S5: Source data of combined enrichment intensities and integration scores for key metabolic pathways; Table S6: Sequences of primers used for qRT-PCR validation of candidate genes.
