# Supplementary figures and images for: Transcriptomic and Metabolomic Profiling Reveals Differential Responses of Soybean Germination to Neutral and Alkaline Salt Stresses
Source: Biology (Basel). 2026 Apr 24;15(9):670. doi: 10.3390/biology15090670 (PMC13162729; doi:10.3390/biology15090670)

PCA

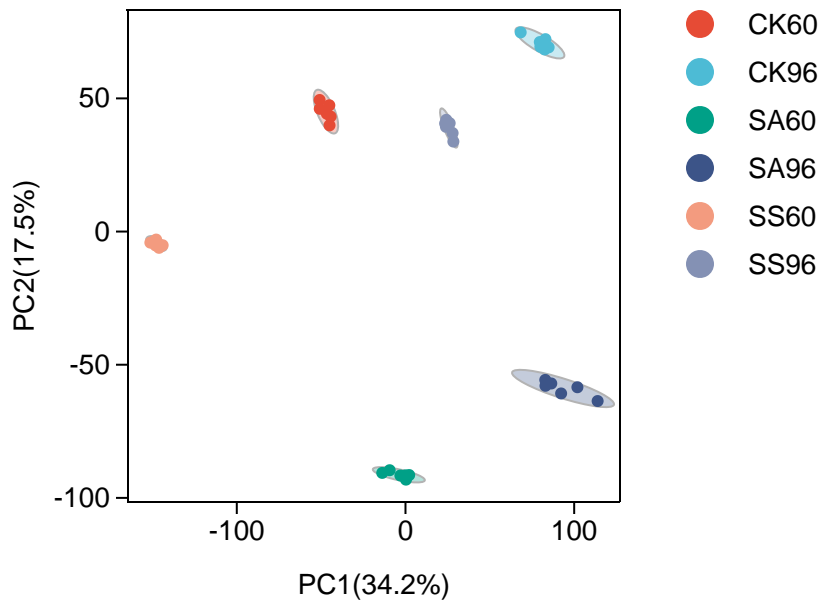

Supplement: Supplementary file 1 [file biology-15-00670-s001.zip › Supplementary Materials/Figure S1 PCA Plot.pdf]

# Correlation Analysis

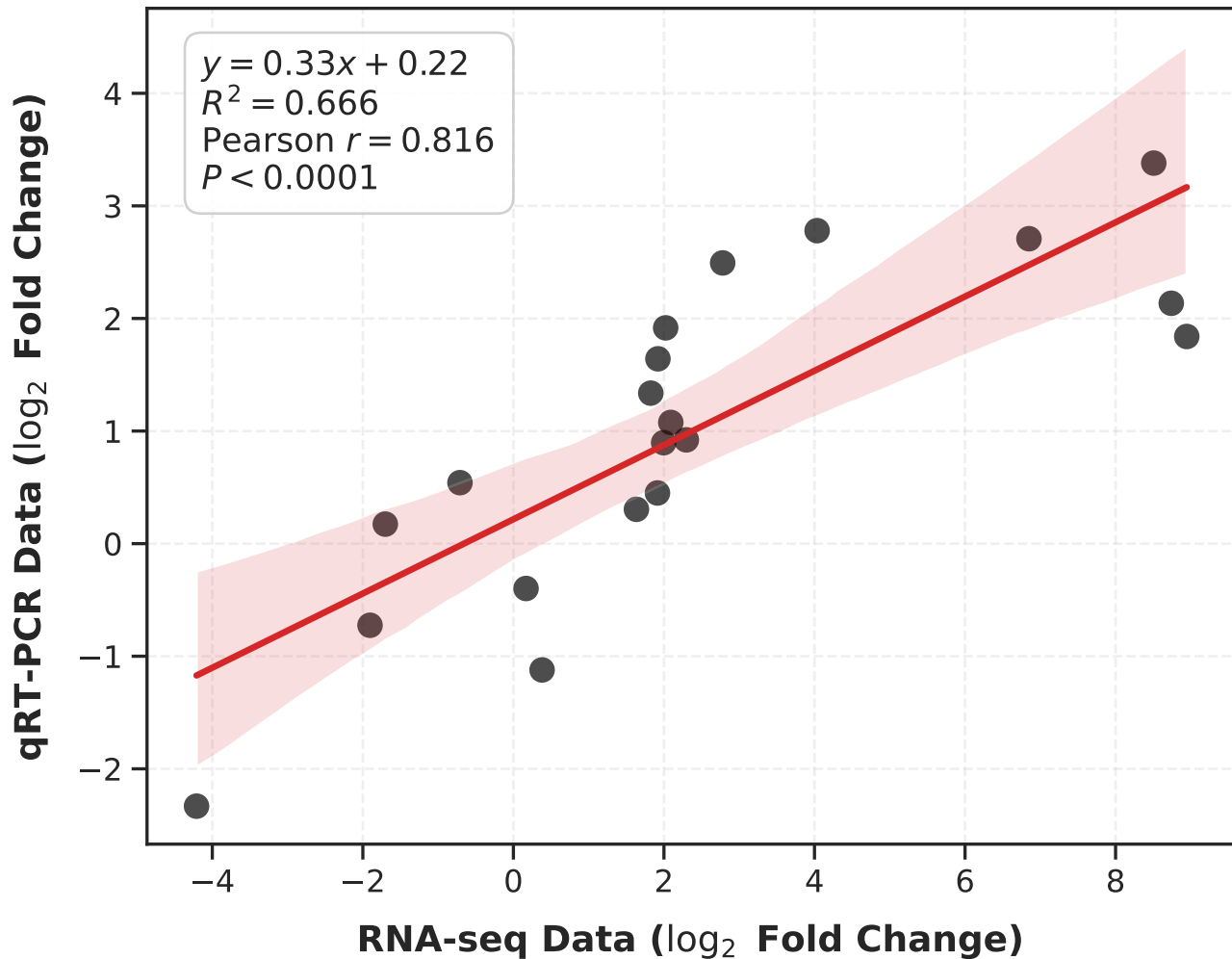

Supplement: Supplementary file 1 [file biology-15-00670-s001.zip › Supplementary Materials/Figure S4 qRTPCR Correlation.pdf]
